# Multi-Mode Love-Wave SAW Magnetic-Field Sensors

**DOI:** 10.3390/s20123421

**Published:** 2020-06-17

**Authors:** Julius Schmalz, Anne Kittmann, Phillip Durdaut, Benjamin Spetzler, Franz Faupel, Michael Höft, Eckhard Quandt, Martina Gerken

**Affiliations:** 1 Integrated Systems and Photonics, Institute of Electrical Engineering and Information Technology, Kiel University, Kaiserstraße 2, 24143 Kiel, Germany; mge@tf.uni-kiel.de; 2 Inorganic Functional Materials, Institute for Materials Science, Kiel University, Kaiserstraße 2, 24143 Kiel, Germany; anki@tf.uni-kiel.de (A.K.); eq@tf.uni-kiel.de (E.Q.); 3 Chair of Microwave Engineering, Institute of Electrical Engineering and Information Technology, Kiel University, Kaiserstraße 2, 24143 Kiel, Germany; pd@tf.uni-kiel.de (P.D.); mh@tf.uni-kiel.de (M.H.); 4 Multicomponent Materials, Institute for Materials Science, Kiel University, Kaiserstraße 2, 24143 Kiel, Germany; besp@tf.uni-kiel.de (B.S.); ff@tf.uni-kiel.de (F.F.)

**Keywords:** SAW, FEM, Love-wave, higher modes, multi-mode, magnetic-field sensing, delta-E effect

## Abstract

A surface-acoustic-wave (SAW) magnetic-field sensor utilizing fundamental, first- and second-order Love-wave modes is investigated. A 4.5 μm SiO_2_ guiding layer on an ST-cut quartz substrate is coated with a 200 nm (Fe_90_Co_10_)_78_Si_12_B_10_ magnetostrictive layer in a delay-line configuration. Love-waves are excited and detected by two interdigital transducers (IDT). The delta-E effect in the magnetostrictive layer causes a phase change with applied magnetic field. A sensitivity of 1250${}^{{}^{\circ}}$/mT is measured for the fundamental Love mode at 263 MHz. For the first-order Love mode a value of 45${}^{{}^{\circ}}$/mT is obtained at 352 MHz. This result is compared to finite-element-method (FEM) simulations using one-dimensional (1D) and two-and-a-half-dimensional (2.5 D) models. The FEM simulations confirm the large drop in sensitivity as the first-order mode is close to cut-off. For multi-mode operation, we identify as a suitable geometry a guiding layer to wavelength ratio of $h_{\mathrm{GL}}/\lambda \approx 1.5$ for an IDT pitch of p=12 μm. For this layer configuration, the first three modes are sufficiently far away from cut-off and show good sensitivity.

## 1. Introduction

**Figure 1:**
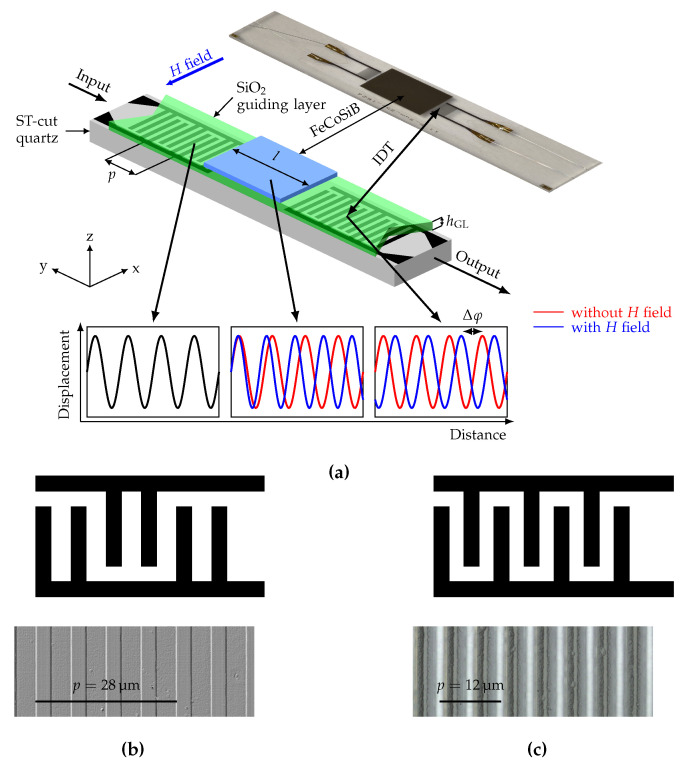
(**a**) Sensor schematic of a SAW-based magnetic-field delay-line sensor and photograph of fabricated sensor including the materials of the different layers and important design parameters. Microscope image and schematic overview of the interdigital transducer (IDT) structure (**b**) for a 28 μm pitch with double-finger electrodes and (**c**) for a 12 μm pitch with single-finger electrodes. The experimental data in this study is based on the 12 μm IDT pitch design. The 28 μm design is included for comparison and described in more detail in [5].

Within the last 30 years, Love-wave-based SAW sensors have been proposed for (bio)chemical measurements [1,2,3]. These sensors use shear-wave surface modes (Love-waves) supported by a guiding layer. Recently, Love-wave SAW sensors have been developed for highly sensitive magnetic-field sensing [4,5,6] using either resonant [7,8] or delay-line structures [5,9,10,11]. In Love-wave magnetic-field sensors, the delay line of the SAW device is coated with a magnetostrictive (MS) layer as shown in Figure 1. The propagation delay line is between two interdigital electrodes (IDT), a transmitter and a receiver electrode. The wavelength of the wave generated at the transmitter IDT is given by the IDT finger pitch *p*, while the wave velocity depends on the layer thicknesses and material parameters. Due to the different layer stack of the delay-line region with the MS layer, the wave velocity and the wavelength decrease, before returning to its original velocity and wavelength at the receiver IDT. Applying a magnetic field changes the effective mechanical stiffness of the MS material via the delta-E effect [12,13,14,15]. This results in a change of the wave velocity and thereby in a magnetic field dependent phase change Δφ as illustrated in Figure 1. From the phase change Δφ the magnetic-field strength can be calculated. Besides other SAW sensors [5,6,7,16,17,18], the delta-E effect is used for magnetic-field sensing with magnetoelectric composite resonators operating in bending or bulk modes [19,20,21,22]. In contrast to such sensors, the sensitivity of Love-wave magnetic-field sensors is expected to benefit from shear-resonance, as the elastic modulus change of the magnetostrictive material was reported to be largest for shear stress [14]. A focus of current research is to maximize the sensitivity, which is essential to overcome thermal phase noise, thus allowing measurements of small amplitude magnetic fields. An increase in sensitivity can be achieved, e.g., with smaller wavelengths by adjusting the sensor geometry. With decreased wavelength and thus increased frequency, the wave energy concentrates further at the surface. By this, higher energies within the MS layer result in increased sensitivity, as the influence on the wave velocity of a magnetically induced elastic modulus change is increased. Because changing the sensor geometry implies production of new sensor types, modifications of existing sensors in terms of excitation is beneficial to increase the sensitivity. Higher harmonics can be used to excite waves with smaller wavelengths resulting in an increase in velocity variation [23]. As the sensitivity rises with decreased wavelength and increased frequency, an alternative approach is to investigate higher order Love-modes, optionally combined with multi-mode operation, to maximize the sensor sensitivity. In the present study, we analyze whether the use of higher order Love-modes increases the sensitivity and if multi-mode operation is possible with this approach. Here, we experimentally demonstrate magnetic-field sensor operation in the fundamental (0th order) mode and in the 1st order mode of a single sensor device and investigate the respective sensitivities. Furthermore, we compare the experimental results to finite-element-method (FEM) simulations of the 0th, 1st, and 2nd order mode. The results are of high importance for the development of SAW sensors for multi-mode operation.

## 2. Experimental Data

For sensor fabrication, first interdigital transducer (IDT) electrodes are structured by ion-beam etching on a 500 μm ST-cut quartz substrate. The electrodes consist of 300 nm Au with 12 nm Cr adhesion layer to the substrate and to the guiding layer on top and are deposited via magnetron sputtering onto the quartz substrate. The sputter deposition rate is determined for all layers prior final sensor fabrication by profilometry. The IDT electrodes have 25 pairs in a single-finger structure with a periodicity of 12 μm and a finger width of 3 μm. For comparison, IDTs with 25 pairs of double-finger electrodes with a periodicity of 28 μm are used. Apart from the IDT structure, the fabrication process of the sensors is identical. The propagation direction is aligned orthogonal to the *x*-axis of the ST-cut quartz wafer for shear-wave excitation. A 4.5 μm thick SiO_2_ guiding layer is deposited with a PECVD process covering the IDT electrodes. Finally, a 200 nm (Fe_90_Co_10_)_78_Si_12_B_10_ (FeCoSiB) magnetostrictive layer is deposited by magnetron sputtering from a single target with composition of (Fe_90_Co_10_)_78_Si_12_B_10_ and structured using a lift-off process [24] to yield the geometry shown in Figure 1. To promote adhesion and prevent oxidation, 10 nm Ta layers on both sides of the FeCoSiB layer were deposited. During deposition, a magnetic field is applied along the *y*-axis to saturate the film and introduce an easy axis of magnetization [11]. The total stack thicknesses for IDTs and MS layer (Cr/Au/Cr and Ta/FeCoSiB/Ta) are controlled by profilometer after structuring. The IDT pitch is chosen supporting the fundamental Love-wave mode and the 1st order mode in the guiding layer with 4.5 µm thickness. This permits excitation of both modes in the same device, while keeping all other material and geometry parameters the same. To achieve this, the IDT pitch and thus the wavelength need to be chosen sufficiently small to enable the wave guide to support higher order Love-wave modes. Hence, the smallest IDT pitch according to the manufacturing limits p=4 μm was chosen and the presence of the 1st mode for this was verified using the 2.5D FEM model described in Section 3.

**Figure 2:**
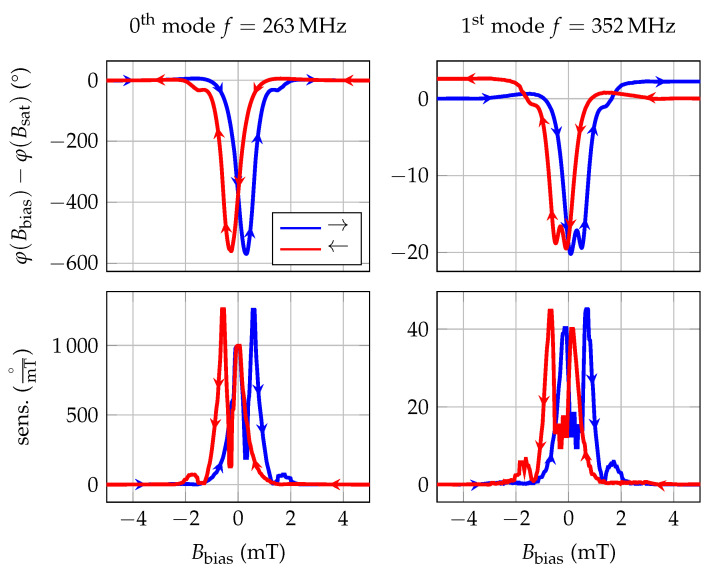
Experimental phase change and sensitivity derived from phase change for 0th and 1st order mode of a delay-line SAW sensor with IDT pitch p=12 μm, guiding layer thickness $h_{\mathrm{GL}}=4.5\;\mu m$ and magnetostrictive layer thickness $h_{\mathrm{MS}}=200\;nm$. The arrow marks represent the direction of the magnetization process starting from a magnetically saturated state. The phase of the sensor in the magnetically saturated state is chosen as phase reference for the measurement.

For the determination of the magnetically induced phase shift, the sensor is placed in a homogeneous magnetic field of a solenoid, which in turn is placed in a magnetic-field shielding mu-metal cylinder ZG1 (Aaronia AG) to avoid significant offsets due to the earth’s magnetic field. The calibrated solenoid is driven by a programmable current source (KEPCO BOP20-10ML) to successively generate magnetic flux densities $B_{\mathrm{bias}}$ between negative and positive saturation. Due to the expected hysteresis of the magnetic material, the measurement is performed for increasing ambient fields from −5 mT to 5 mT and vice versa. The static phase response of the sensor is determined with a lock-in amplifier UHFLI (Zurich Instruments) at a sensor input power of 0 m. The resulting phase responses are shown in Figure 2, in which blue lines correspond to a change of the magnetic flux density from negative to positive values and red lines from positive to negative values. Due to the significant group delay of the sensor > 1 μs, a single-frequency measurement of the phase leads to an ambiguity of n·2π. Therefore, the depicted phase responses (Figure 2) are each normalized to the value in magnetic saturation. The measured phase shift results from the delta-E effect, dominated here by the change of the shear modulus. The bias field dependent sensitivity *S* is given by the derivative of the measured phase change (Figure 2). The fundamental mode is at 263 MHz, exhibiting a maximum sensitivity of 1250${}^{{}^{\circ}}$/mT at a bias field of 0.6 mT. The 1st order mode is at 352 MHz and has a much lower maximum sensitivity of only 45${}^{{}^{\circ}}$/mT at a bias field of 0.7 mT. To explain this strong reduction in sensitivity, finite-element-method (FEM) simulations were carried out.

The sensitivity of the fundamental mode measured here is only outperformed by the sensors described by Schell et al. [11] with 2000${}^{{}^{\circ}}$/mT using a magnetic anisotropy controlled magnetostrictive layer or sensors with higher MS layer thicknesses [24]. Potentially, using these more enhanced layers, the overall sensitivity of the sensor geometry in this study could even surpass the one described there due to its enhanced magnetic sensitivity. In the literature, various sensor types with lower performance have been described. Ganguly et al. [25] and Robbins and Simpson [26] started the development of SAW-based magnetically tunable phase shifters and did not provide a sensitivity value. To be able to classify their devices anyway, the sensitivities of up to 5${}^{{}^{\circ}}$/mT were extracted from the phase responses. Forester et al. [9] reported an enhanced SAW device with to 240${}^{{}^{\circ}}$/mT (value extracted from phase response), while Li et al. [27] describe the performance by giving the maximum phase change of 0.64% and Yokokawa et al. [4] mention a phase shift normalized to the delay-line length of 1500${}^{{}^{\circ}}$/cm. With 6944${}^{{}^{\circ}}$/cm, the sensitivity of the sensors described here is significantly higher using a delay-line length of 1.8 mm. Hanna [16] and Wang et al. [28] describe the limit of detection (LOD) of their sensors to be 1 μT and 140 nT, respectively. As only the sensor dimension and the frequency is changed and the same layer thicknesses and material deposition processes were used, the sensor investigated here with p=12 μm can be expected to have a similar LOD than the sensor described in a previous study with $250pT/\sqrt{\mathrm{Hz}}$ at 10 Hz [5]. Nevertheless, the main problem remains that the comparison of these sensors using different surface waves, materials, geometries and ambiguous specifications of the sensor performance is difficult. However, within this study, the sensitivity measured in ${}^{{}^{\circ}}/mT$ is used for comparison, as this value can be specified for the measurement, as well as for our FEM simulations.

For completeness, the limit of detection results from this sensor are shown in Figure A2 in the Appendix C, as the focus of this study is based on sensitivities. The fundamental mode reaches an LOD of $700pT/\sqrt{\mathrm{Hz}}$ and the first mode $3nT/\sqrt{\mathrm{Hz}}$ both at 10 Hz.

As the noise increases with the sensitivity due to the magnetic layer properties (see Appendix C), an LOD-improvement of the sensors described here was not observed. However, the improvement of the sensor sensitivity will be beneficial for the development of future sensors with more sophisticated magnetic layers.

## 3. FEM Modelling

For FEM simulations, 1D and 2.5D unit cell models were implemented as described in [29] and solved using Comsol Multiphysics^®^. For the 1D model, the geometry of the device is reduced to a stack of layers with different thicknesses assuming isotropic material parameters. By this, the displacement $u_{1}$ in *x*-direction is only *z*-dependent and is described by

(1) $$\begin{array}{c} u_{1}\left(k,z,\omega \right)=U\left(k,z,\omega \right)e^{i(ky-\omega t)} \end{array}$$

with the wave vector *k*, the time *t* and the angular frequency ω. After transformations and substitutions, the differential equation for Love-waves [30] is obtained:

(2) $$\begin{array}{c} \frac{d}{dz}\left[G\left(z\right)\frac{dU}{dz}\right]=\left[k^{2}G\left(z\right)-\omega^{2}\rho \left(z\right)\right]U\left(k,z,\omega \right). \end{array}$$

with the shear modulus *G* and the density ρ. This equation is solved using a partial differential equation interface in Comsol Multiphysics^®^ with the following boundary conditions: The bottom interface is clamped (displacement $u_{1}=0$) and the top interface is a free surface (stress $T_{13}=0$). At the layer interfaces, continuity of displacement and stress is required. The stress component $T_{13}$ must be continuous at the interfaces of the different layers, which is executed automatically by the program.

**Figure 3:**
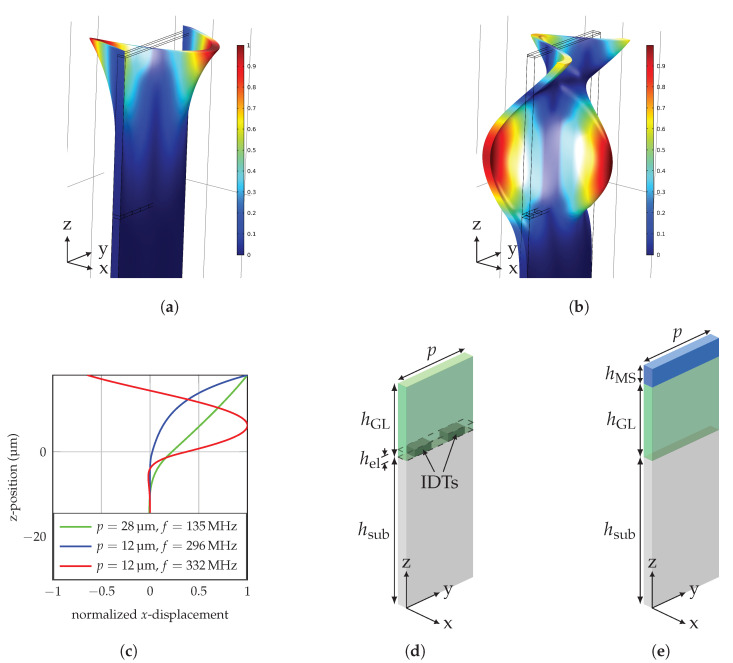
Normalized displacement of a delay-line SAW device with λ=12 μm and $h_{\mathrm{GL}}=4.5\;\mu m$ for the (**a**) 0th mode and (**b**) 1st mode calculated using a requency domain study. (**c**) Mode profile of Love-waves at different wavelengths with a 500 μm thick quartz substrate for z<0 and a 18 μm thick guiding layer above z=0. Schematic simulation setup of the unit cell for the excitation region (**d**) and the delay-line region (**e**). With a width of *p*, substrate thickness $h_{\mathrm{sub}}$, guiding layer thickness $h_{\mathrm{GL}}$, electrode thickness $h_{\mathrm{el}}$, magnetostrictive layer thickness $h_{\mathrm{MS}}$ and the interdigital transducers (IDTs).

The 2.5D simulation solves the 3-dimensional problem assuming that the stress and strain distributions do not depend on the *x*-coordinate and no stress in *x*-direction, using a so-called plain-strain conditions. As the width of our device is large compared to the wavelength, these conditions can be assumed. In *y*-direction periodic boundary conditions are used. The 2.5D model reduces the amount of mesh knots and therefore the computational time significantly in contrast to a full 3D model. The used material parameters for both models are described in Appendix A and the delta-E effect is represented by a linear approximation at the sensor operating point. A linear approximation of the delta-E effect was considered, because the targeted magnetic fields are significantly low and thus, a small signal approximation is suitable. While the 1D model only considers different layer thickness using isotropic material parameters, the 2.5D model additionally includes the IDT structure as shown in Figure 3d and uses anisotropic material parameters to obtain more realistic results.

The simulation setup consists of an ST-cut quartz substrate with a thickness of $h_{\mathrm{sub}}=200\;\mu m$, a guiding layer of varying thickness $h_{\mathrm{GL}}$ and a magnetostrictive layer with a thickness of $h_{\mathrm{MS}}=200\;nm$. The electrode thickness $h_{\mathrm{el}}$ is 300 nm. The wave is excited using single-finger IDTs at the top of the substrate by applying an electric potential.

### 3.1. Mode Profile

The mode profiles showing the amplitude of the displacement for all positions *z* in the layer are evaluated for IDT pitches of 12 μm and 28 μm. The IDT pitch corresponds to the mode wavelength. Figure 3a,b show the displacement profiles in the x-direction for 0th and 1st order Love-wave modes propagating along the y-direction for a pitch of 12 μm obtained with the 2.5D model. From the graphs, the displacement profiles shown in Figure 3c are extracted. For comparison, the fundamental mode for a pitch of 28 μm is additionally shown. Only the top 200 nm region consists of magnetostrictive material, while the guiding layer has a thickness of 18 μm in Figure 3. This guiding layer thickness was chosen for better visibility of the wave concentration effect.

Of the three modes, the 0th mode at p=12 μm has the highest concentration at the surface, because the largest fraction of the area between the displacement graph and the zero line is localized in the MS layer. For the 1st order mode at 332 MHz, a large part of the displacement is in the guiding layer and not only at the surface. Thus, a significantly reduced sensitivity is expected already from this qualitative analysis.

### 3.2. Two-Part Simulation

Since the Love-wave is generated at the IDT without a magnetostrictive layer on top before propagating through the delay line, the simulation must take into account the altered layer structure with the magnetostrictive layer. This is done by dividing the simulation into two parts: the wave excitation and propagation. Neglecting the changed layer structure by using the same wavelength for both simulations parts, with and without an MS layer, respectively, would lead to a drastic frequency shift and thus, distort sensitivity results. The frequency of a sensor with $h_{\mathrm{GL}}=4.5\;\mu m$ is, e.g., reduced from 329 MHz without an MS layer to 300 MHz with an MS layer of 200 nm thickness. To avoid the influence of this effect on the sensitivity, we implemented the two-part study.

In the excitation region (Figure 3d), the wavelength is given by the IDT pitch, as the waves are constructively interfering with a periodicity of *p*. The resonance frequency is given by the different layer thicknesses and their properties. Hence, the first part calculates the mode frequency for the given guiding layer thickness without an MS layer, but with IDTs to address the wave’s excitation at the IDT structure. This is achieved by a frequency sweep using a frequency domain study and the 2.5D model. The resonance frequency is identified, where the displacement of the Love-wave is maximized.

After the excitation, the wave is propagating through the delay line with an MS layer on top and without IDTs (Figure 3e). This change in the layer stack leads to an altered wavelength of the Love-wave. Since the frequency of the wave cannot change after excitation, it is set to the resonance frequency of the first part. Hence, the second part uses the excitation frequency from the first part to calculate the influence of the changed layer stack. Using unit cell models, this is only possible using a sweep, until the unit cell width fits to the mode’s wavelength at that given frequency. In particular, a frequency domain study is carried out for unit cell widths near the expected wavelength and the resonance wavelength is identified at the point of maximum displacement.

### 3.3. 1D vs. 2.5D

**Figure 4:**
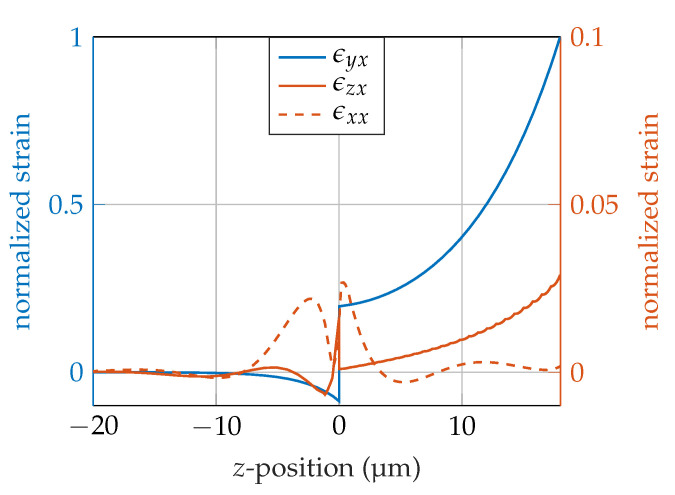
Comparison of strain tensor components of a SAW sensor at the first mode with 18 μm guiding layer thickness and substrate for z < 0. The components are normalized to the maximum of the main shear component $\epsilon_{yx}$. The magnitude of the components $\epsilon_{zx}$ and $\epsilon_{xx}$ are shown on the right axis.

For model verification, we compared the 1D and 2.5D results. The 1D model uses isotropic material parameters. Therefore, we used an isotropic material tensor for the 2.5D simulation. This enables a comparison of the models using the same material parameters. For comparison, the resonance frequency from the 1D model is used in the 2.5D model. Comparing the 1D model with the 2.5D model with isotropic material parameters, reveals a deviation between the two models of <0.2 for our parameter sets (results exemplary shown for p=12 μm and $h_{\mathrm{GL}}=18\;\mu m$ for the 0th and 1st order mode, Table 1). Comparing the 2.5D model with isotropic material parameters with the same model with anisotropic material parameters, there is a deviation <2%. The wavelength as well as the wave velocity are clearly reduced, as the wave is not at the IDT resonance wavelength. At the resonance frequency of 295 MHz, the wavelength reaches approximately 12 μm, again. The anisotropic material parameters used in the 2.5D model cause a frequency shift in the resonance frequency and there are other strain components than the targeted shear component $\epsilon_{yx}$ caused by the anisotropy of the material parameters (Figure 4).

## 4. Sensitivity Calculation

With the use of the FEM models, the influence of the concentration of the energy at the surface on the sensitivity is investigated. In a previous study, we showed that the sensitivity *S* of the present SAW sensors can be separated into three parts: the magnetic sensitivity $S_{\mathrm{mag}}$, the structural sensitivity $S_{\mathrm{str}}$ and the geometrical sensitivity $S_{\mathrm{geo}}$ [5]. The sensitivity *S* is calculated using Equation (3) with the shear modulus *G* and the magnetic field *H*.

(3) $$S=\frac{\partial \varphi }{\partial H}=\frac{\partial G}{\partial H}\;\cdot \;\frac{\partial v}{\partial G}\;\cdot \;\frac{\partial \varphi }{\partial v}=S_{\mathrm{mag}}\;\cdot \;S_{\mathrm{str}}\;\cdot \;S_{\mathrm{geo}}$$

The magnetic sensitivity $S_{\mathrm{mag}}$ describes the shear modulus change for a given magnetic-field change and depends on the magnetic properties of the material. It is also highly dependent on the bias magnetic-field $B_{\mathrm{bias}}$, which is chosen to get maximal shear modulus change. The structural sensitivity $S_{\mathrm{str}}$ describes the amount of wave velocity change per shear modulus change and depends on the amount of wave energy propagating through the MS layer and is mainly determined by the layer structure of the sensor. The geometric sensitivity $S_{\mathrm{geo}}$ describes phase change per velocity change and depends on the excitation frequency and length *l* of the delay line. The magnetic sensitivity $S_{\mathrm{mag}}$ is approximated by Equation (4) at the operating point of 0.6  mT. For this operating point, the bias field $B_{\mathrm{bias}}$ was used, where the sensitivity reaches its maximum. The magnetic material properties were obtained by an iterative fit, until the phase change derived from the measurment and the FEM simulation using the assumed magnetic properties calculated by a micro-magnetic model [22] matched. As this process requires several iterations and the determination of the magnetic parameters is not trivial, this fit was carried out using the 1D and not the 2.5D model because of the faster computation time. The magnetic sensitivity at the operating point with the two shear moduli $G_{\mathrm{MS}}^{+}$ and $G_{\mathrm{MS}}^{-}$ for a magnetic flux density change of ΔB=0.1 mT is given by:

(4) $$S_{\mathrm{mag}}=\frac{G_{\mathrm{MS}}^{+}-G_{\mathrm{MS}}^{-}}{\Delta B}=\frac{29.8605\;G\mathrm{Pa}-26.3380\;G\mathrm{Pa}}{0.1\;mT}=35.225\;\frac{G\mathrm{Pa}}{mT}.$$

The differential quotient of the structural sensitivity $S_{\mathrm{str}}$ is approximated by a difference quotient:

(5) $$S_{\mathrm{str}}=\frac{\partial v}{\partial G}=\frac{f\Delta \lambda }{\Delta G}.$$

For this, two simulations around the operating point G=28.1 GPa are carried out. These two values of the elasticity tensor $c_{\mathrm{MG}}^{EH}$ of the magnetostrictive layer (surrounding the operating point) are $c_{\mathrm{MG}}^{EH+}$ and $c_{\mathrm{MG}}^{EH-}$ being 0.1  mT apart from each other.

For this simulation, the frequency from the first part of the simulation was used to calculate the wave propagation constants. The eigen-wavelength of the mode is calculated for a respective geometry with the material parameters $c_{\mathrm{MG}}^{EH+}$ and $c_{\mathrm{MG}}^{EH-}$ at a given frequency.

The results of this two-stage approach are values for the wavelength and the frequency for the two different points near the operating point. With these, the structural sensitivity is calculated using (5), with the wave velocity v=λf with constant *f* and ΔG=29.8605 GPa−26.3380 GPa. The wave propagates through the delay line with the length l=150 p and according to its wave constant *k*, the phase shift accumulates to φ=kl. With this, the geometric sensitivity is given by Equation (6).

(6) $$S_{\mathrm{geo}}=\frac{\partial \varphi }{\partial v}=\frac{\partial kl}{\partial v}=\frac{\partial }{\partial v}\frac{2\pi fl}{v}=-\frac{2\pi fl}{v^{2}}$$

**Figure 5:**
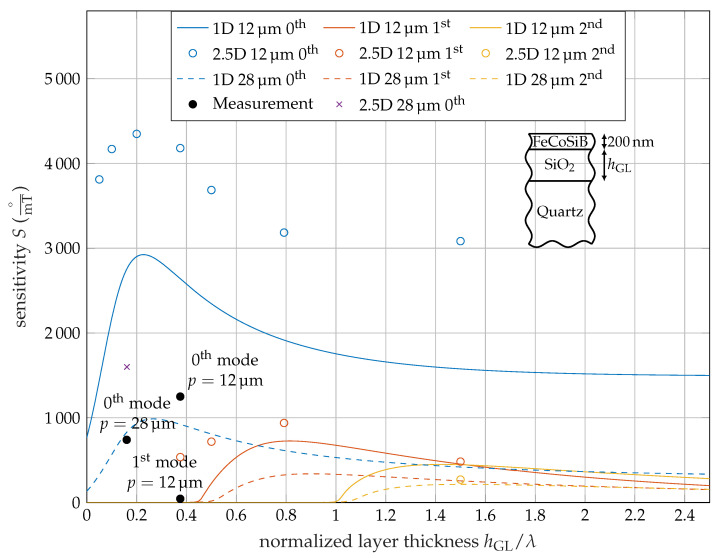
Comparison of sensitivity results for isotropic (1D) and anisotropic (2.5D) calculation for p=12 μm and p=28 μm for the fundamental, first- and second-order modes. Due to the fixed MS layer thickness, the normalized data are not entirely wavelength independent. For comparison, the measurement results are included.

Using these equations, the overall sensitivity is calculated for different guiding layer thicknesses and IDT pitches for the fundamental, 1st, and 2nd mode. The 2.5D simulation results are added for several points in Figure 5, showing the sensitivity for different $h_{\mathrm{GL}}/\lambda$-ratios. For comparison, results from a 1D setup with same layer thicknesses but isotropic material parameters are additionally shown. Similar to the 2.5D simulation described above, the 1D simulation is split into two parts as well including the first part to determine the resonance frequency. In the second part, two simulations near the operating point are carried out using the values $G_{\mathrm{MS}}^{+}$ and $G_{\mathrm{MS}}^{-}$ as the shear modulus of the magnetostrictive layer. In contrast to the 2.5D simulation, there are no parameter sweeps necessary to find the resonances, as the differential equation [29] can be solved directly. The 1D results are shown as continuous lines, since the computation time is a fraction of the 2.5D simulation.

## 5. Results and Discussion

The sensitivity curve for the Love-wave magnetic-field sensors at different $h_{\mathrm{GL}}/\lambda$-ratios is shown in Figure 5 for p=12 μm and p=28 μm. The sensitivity curve starts with an axial intercept for the 0th mode, because even with no guiding layer ($h_{\mathrm{GL}}=0\;\mu m$), there is still a layer of 200 nm thick MS material. Due to the material properties this layer acts as a guiding layer itself and by this supports Love-waves. With increasing guiding layer thickness, the sensitivity reaches a maximum. Here, the highest part of the wave energy is at the surface. Thus, an additional guiding layer improves the performance. With even higher guiding layer thicknesses, the wave starts to concentrate within the guiding layer and less at the surface and the sensitivity decreases.

Above a cut of ratio of $h_{\mathrm{GL}}/\lambda \approx 0.5$ and $h_{\mathrm{GL}}/\lambda \approx 1$ the 1st and 2nd order modes are supported and with further increasing guiding layer thickness, the sensitivity increases to a maximum similar to the fundamental mode (Figure 5).

The overall behavior of less sensitive higher modes is similar to the one published by Kovacs et al [31], where a Love-wave sensor with a silicon substrate and a SiO_2_ guiding layer was examined. The authors described a sensor using the frequency shift due to mass absorption as detection mechanism, which is contrary to the sensor analyzed here using an MS layer causing a phase shift. For the calculation, only isotropic material parameters are used and the higher 2.5D results were not predicted.

### 5.1. Qualitative Results

The 0th order modes sensitivity is significantly larger than the 1st and 2nd order modes sensitivity, as the wave is more concentrated at the surface. Figure 3 confirms this, as the displacement of the 1st order mode at the surface is lower than the maximal displacement resulting in an reduced influence of the MS layer to the wave propagation. Hence, the sensitivity is reduced.

The curve shapes of the 1D and 2.5D simulation results are similar, but with an offset. The overall shape of both the 1D and 2.5D simulations indicates a maximum near $h_{\mathrm{GL}}/\lambda =0.3$, $h_{\mathrm{GL}}/\lambda =0.7$ and $h_{\mathrm{GL}}/\lambda =1.5$ for the fundamental, 1st and 2nd mode. Regarding the isotropic material parameters for the 1D and 2.5D simulation, the results are in good agreement in terms of the propagation constants. The sensitivities match with an error below 3% according to Table 2. Consequently, the only difference between the models is the anisotropy of the quartz substrate. The 2.5D simulation takes more details of the materials into account and the results have a significant offset in the sensitivity. The frequency of the 2.5D simulation with anisotropic material parameters is lower than the isotropic results. This emphasizes the importance of using 2.5D models to get higher accuracy of SAW magnetic-field sensors.

According to Figure 5, multi-mode operation of the fundamental, 1st, and 2nd modes is possible and a ratio of $h_{\mathrm{GL}}/\lambda =1.5$ for p=12 μm is preferred, because all three modes have a reasonable high sensitivity level. Regarding overall sensitivity, the choice of the operating point is not optimal, since the fundamental mode’s sensitivity is not at its maximum. Nevertheless, it still surpasses the maximal sensitivity of a sensor with p=28 μm and by this facilitates multi-mode operation.

### 5.2. Quantitative Results

Because the 1D and the 2.5D simulation uses the same values for the magnetic sensitivity according to Equation (4), which was fit to the 1D model, the 1D simulation results correspond better to the experimental data than the 2.5D simulation, especially the fundamental mode.

A surprisingly strong reduction of the 1st order modes sensitivity in the measurement was observed. The FEM simulation confirmed an overall lower performance of the higher modes, although the experimental values were even lower. This data point for the 1st order mode is extremely close to the cut-off ratio. Due to the steep curve near the cut-off ratio, small deviations between measurement and simulation can cause a significant influence on the sensitivity. One effect not considered in the simulation is the frequency dependency of the delta-E effect that might result in a slightly reduced magnetic sensitivity in higher frequency modes according to theoretical considerations [22].

**Figure A1:**
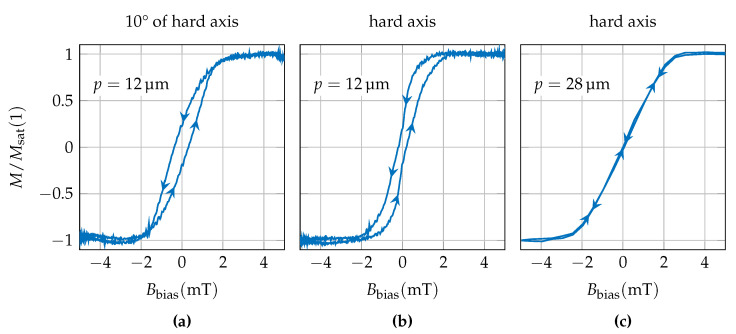
Magnetization curve of the SAW device with p=12 μm measured by a hysteresis loop tracer model 108 (Shb Instruments). The normalized magnetization parallel to the delay line (**a**) and to the hard axis (**b**) are shown. (**c**) Shows the Magnetization curve of a SAW device with p=28 μm [5]. The arrow marks represent the direction of the magnetization process starting from a magnetically saturated state.

**Figure A2:**
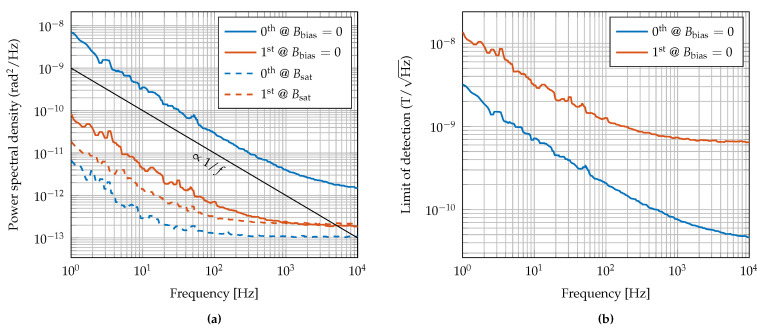
(**a**) Measured power spectral densities of random phase fluctuations in the magnetic operating point (continuous lines) and in magnetic saturation (dashed line) of the sensor with p=12 μm for fundamental and first-order mode. (**b**) Calculated limit of detection for both modes.

Additionally, a lower $S_{\mathrm{mag}}$ due to fabrication variations, internal stress and material inhomogeneities probably causes the lower performance of the measurement in general, as even the fundamental modes sensitivity is lower than in the simulation. Because $S_{\mathrm{mag}}$ of the sensor with p=28 μm was used in the simulation a difference between simulation and measurement is expected for p=12 μm. The magnetization curves (Figure A1) of the sensor with p=12 μm show a significant tilt of the mean easy axis (Figure A1a) compared to the sensor with p=28 μm (Figure A1c) [5]. It also exhibits a higher coercivity along the hard axis that might result from inhomogeneous magnetic anisotropies or shape effects. Both the easy axis tilt and the inhomogeneity are expected to reduce the delta-E effect and thereby $S_{\mathrm{mag}}$ [32,33,34].

## 6. Conclusions

We investigated a Love-wave-based magnetic-field sensor operated in different modes in experiment and simulation. Our fabricated sensor with p=12 μm show a 250 higher sensitivity compared to p=28 μm for the fundamental mode, corresponding to the prediction from [5]. The significant reduction of the first mode sensitivity is explained with the generally reduced sensitivity of the higher modes according to Figure 5 caused by the easy axis tilt and increased mean anisotropy (Figure A1) and in particular with this mode being close to cut-off. We also showed the necessity to use more detailed FEM models, as the one-dimensional model does not satisfy the anisotropy of the used materials and excitation effects due to the electrodes. This results in significant deviations, hence this model can only be used as an approximation.

We proposed a sensor with $h_{\mathrm{GL}}/\lambda =1.5$ for multi-mode operation possessing fundamental, 1st, and 2nd order Love-wave modes with sufficient sensitivity. Although the higher-mode sensitivity is generally reduced compared to the fundamental mode, the choice of this operating point enables the reduction of noise and distortions using signal processing approaches with mode- instead of time-domain averaging of the signal. Assuming an equal sensitivity of each mode, the sensitivity increases linearly with the number of averaged modes [35]. For non-identical values, the overall sensitivity is given by the sum of the individual sensitivities. Thus, for the proposed multi-mode operating point, the combined sensitivity is not increasing linearly with the number of modes, but is a result of the addition of the individual sensitivities. Since almost the entire wave energy is trapped in the guiding layer for higher $h_{\mathrm{GL}}$, the material choice of the guiding layer is important in terms of loss minimization.

## Figures and Tables

**Table 1:** Comparison of results from 1D and 2.5D model with isotropic parameters, 2.5D model with anisotropic parameters for p=12 μm and $h_{\mathrm{GL}}=18\;\mu m$ for the 0th and 1st order mode.

	0th			1st		
	*p*/μm	*f*/MHz	*v*/m/s	*p*/μm	*f*/MHz	*v*/m/s
1D isotropic	12.000	304.1	3648.0	12.000	336.7	4040.0
2.5D isotropic	12.012	304.1	3652.8	12.019	336.7	4046.8
2.5D anisotropic	11.682	304.1	3552.5	11.826	336.7	3981.8
2.5D anisotropic resonant	12.022	295.0	3546.5	11.995	332.0	3982.3

**Table 2:** Comparison of the measured and simulated sensitivities for different wavelengths λ and guiding layer thicknesses $h_{\mathrm{GL}}$.

			0th		1st	
λ/μm	$h_{\mathrm{GL}}$/μm		*f*/MHz	*S*/${}^{{}^{\circ}}/mT$	*f*/MHz	*S*/${}^{{}^{\circ}}/mT$
28	4.5	measurement	148	740	−	−
		1D isotropic	162	750	−	−
		2.5D anisotropic	150	1600	−	−
12	4.5	measurement	263	1250	352	45
		1D isotropic	320	2640	420	0.6
		2.5D anisotropic	304	4180	368	530
	9.5	1D isotropic	306	1916	382	725
		2.5D anisotropic	297	3184	368	938
	18	1D isotropic	304	1574	336	453
		2.5D isotropic	304	1534	336	449
		2.5D anisotropic	295	3084	332	483

## Appendix A. Material Parameters

### Appendix A.1. For One-Dimensional Model

$G_{\mathrm{Quartz}}=67.50\;G\mathrm{Pa}$, $\rho_{\mathrm{Quartz}}=2200\;kg/m^{3}$,

$G_{\mathrm{Si}O_{2}}=33.35\;G\mathrm{Pa}$, $\rho_{\mathrm{Si}O_{2}}=2200\;kg/m^{3}$,

$G_{\mathrm{MS}}^{+}=29.8605\;G\mathrm{Pa}$, $G_{\mathrm{MS}}^{-}=26.3380\;G\mathrm{Pa}$, and $\rho_{\mathrm{MS}}=7250\;kg/m^{3}$.

### Appendix A.2. For 2.5D and 3D

ST-cut quartz (SiO_2_) rotated from [36]:

$c_{\mathrm{Quartz}}^{EH}=\left(\begin{array}{cccccc} 86.74 & 27.49 & -8.60 & 1.05 & 0.00 & 0.00\\ 27.49 & 96.64 & -4.81 & 13.44 & 0.00 & 0.00\\ -8.60 & -4.81 & 130.74 & -1.84 & 0.00 & 0.00\\ 1.05 & 13.44 & -1.84 & 41.22 & 0.00 & 0.00\\ 0.00 & 0.00 & 0.00 & 0.00 & 30.35 & 7.58\\ 0.00 & 0.00 & 0.00 & 0.00 & 7.58 & 67.50 \end{array}\right)\;G\mathrm{Pa}$,

$e_{\mathrm{Quartz}}=\left(\begin{array}{cccccc} 0.17 & -0.04 & -0.13 & 0.08 & 0.00 & 0.00\\ 0.00 & 0.00 & 0.00 & 0.00 & 0.07 & -0.10\\ 0.00 & 0.00 & 0.00 & 0.00 & -0.07 & 0.11 \end{array}\right)\;N/Vm$,

$\varepsilon_{r},\mathrm{Quartz}=\left(\begin{array}{ccc} 4.43 & 0.00 & 0.00\\ 0.00 & 4.54 & 0.10\\ 0.00 & 0.10 & 4.52 \end{array}\right)$,

$\rho_{\mathrm{Quartz}}=2651\;\;kg/m^{3}$.

Silica glass (SiO_2_) [37]:

$c_{\begin{array}{c} \mathrm{SiO}2 \end{array}}^{EH}=\left(\begin{array}{cccccc} 77.46 & 15.6 & 15.6 & 0 & 0 & 0\\ 15.6 & 77.46 & 15.6 & 0 & 0 & 0\\ 15.6 & 15.6 & 77.46 & 0 & 0 & 0\\ 0 & 0 & 0 & 30.91 & 0 & 0\\ 0 & 0 & 0 & 0 & 30.91 & 0\\ 0 & 0 & 0 & 0 & 0 & 30.91 \end{array}\right)\;G\mathrm{Pa}$,

$e_{\begin{array}{c} \mathrm{SiO}2 \end{array}}=\left(\begin{array}{cccccc} 0 & 0 & 0 & 0 & 0 & 0\\ 0 & 0 & 0 & 0 & 0 & 0\\ 0 & 0 & 0 & 0 & 0 & 0 \end{array}\right)\;N/Vm$,

$\varepsilon_{r},\begin{array}{c} \mathrm{SiO}2 \end{array}=\left(\begin{array}{ccc} 2.2 & 0.0 & 0.0\\ 0.0 & 2.2 & 0.0\\ 0.0 & 0.0 & 2.2 \end{array}\right)$,

$\rho_{\mathrm{Si}O_{2}}=2200\;\;kg/m^{3}$.

FeCoSiB [38]:

$c_{\mathrm{MG}}^{EH+}=\left(\begin{array}{cccccc} 104.512 & 44.7907 & 44.7907 & 0 & 0 & 0\\ 44.7907 & 104.512 & 44.7907 & 0 & 0 & 0\\ 44.7907 & 44.7907 & 104.512 & 0 & 0 & 0\\ 0 & 0 & 0 & 29.8605 & 0 & 0\\ 0 & 0 & 0 & 0 & 29.8605 & 0\\ 0 & 0 & 0 & 0 & 0 & 29.8605 \end{array}\right)\;G\mathrm{Pa}$,

$c_{\mathrm{MG}}^{EH-}=\left(\begin{array}{cccccc} 92.1830 & 39.5070 & 39.5070 & 0 & 0 & 0\\ 39.5070 & 92.1830 & 39.5070 & 0 & 0 & 0\\ 39.5070 & 39.5070 & 92.1830 & 0 & 0 & 0\\ 0 & 0 & 0 & 26.3380 & 0 & 0\\ 0 & 0 & 0 & 0 & 26.3380 & 0\\ 0 & 0 & 0 & 0 & 0 & 26.3380 \end{array}\right)\;G\mathrm{Pa}$,

$e_{\mathrm{MG}}=\left(\begin{array}{cccccc} 0 & 0 & 0 & 0 & 0 & 0\\ 0 & 0 & 0 & 0 & 0 & 0\\ 0 & 0 & 0 & 0 & 0 & 0 \end{array}\right)\;N/Vm$,

$\varepsilon_{r},\mathrm{MG}=\left(\begin{array}{ccc} 1 & 0 & 0\\ 0 & 1 & 0\\ 0 & 0 & 1 \end{array}\right)$,

$\rho_{\mathrm{MG}}=7250\;\;kg/m^{3}$.

## Appendix B. B-H Loops

The magnetization curve of the SAW device with p=12 μm are measured by a hysteresis loop tracer model 108 (Shb Instruments). The anisotropy is tilted by approximately 10° with respect to the propagation direction. The normalized magnetization parallel to the delay line is shown in Figure A1a and the normalized magnetization of the hard axis is depicted in Figure A1b. Figure A1c shows the magnetization curve of a SAW device with p=28 μm [5]. A small opening of the hysteresis curve can be seen, which is also visible in the phase change and sensitivity curves plotted in Figure 2.

## Appendix C. Limit of Detection and Phase Noise Measurements

The frequency dependent noise floor of the SAW magnetic-field sensor in units of $T/\sqrt{\mathrm{Hz}}$, often referred to as equivalent magnetic noise floor, detectivity, or limit of detection (LOD) is given by the ratio of the square root of the power spectral density of the sensor’s random phase fluctuations in units of ${\mathrm{rad}}^{2}/\mathrm{Hz}$ and the (phase) sensitivity in units of rad/T [39]. For the first two discussed modes of the sensor under investigation the power spectral densities of the sensor’s random phase fluctuations were measured using a phase noise analyzer FSWP (Rohde & Schwarz) at an input power of 10 dBm m. During these measurements, the sensor was placed inside an electrically, magnetically (ZG1 from Aaronia AG), and acoustically shielded measurement chamber. For each mode, the noise measurements were performed twice: one measurement in the sensor’s magnetic operating point and one measurement in magnetic saturation using a strong permanent magnet. The magnetic operating point was chosen at $B_{\mathrm{bias}}=0$. Compared to maximum possible sensitivities (Figure 2), this operating point is associated with only slightly reduced values for the sensitivities but offers the great advantage that during noise measurements, no additional, and with regard to its additional noise extremely critical, current source is required to generate the magnetic bias flux density. To ensure a largely comparable magnetic operating point for both modes, the sensor was magnetically initialized before each measurement. Using a coil surrounding the sensor and a current source (B2962A from Keysight), the magnetic bias flux density was gradually increased from −10  mT to 0 mT over a period of about 15 s. Subsequently, the current source was switched off before the actual noise measurement was performed. Due to high mismatch losses and corresponding high insertion losses of about 55 dB to 65 dB, two amplifiers (ZFL-1000LN+ from Mini-Circuits) with a total gain of 50 dB were additionally connected between the sensor and the input of the phase noise analyzer. Contrary to the determined sensitivities based on numerically differentiating the static phase responses (Figure 2), values for the sensitivities necessary for calculating the LOD were re-determined in the chosen magnetic operating point of $B_{\mathrm{bias}}=0$ by means of a dynamic sensitivity measurement as described in [24]. Values of $26.2\;\mathrm{rad}/mT=1504\;{}^{{}^{\circ}}/mT$ (0th mode) and $0.67\;\mathrm{rad}/mT=38.2\;{}^{{}^{\circ}}/mT$ (1st mode) were achieved. The results of the phase noise measurements are shown in Figure A2a. As already observed in a previous study [39], at low frequencies at which the thermal noise, i.e., the white phase noise, is negligible the power spectral density not only progresses proportional to 1/f but is also higher than for the magnetically saturated device indicating a magnetic origin for the increased noise. Recently, it was found [24] that the 1/f flicker phase noise in the sensor’s magnetic operating point is associated with hysteresis losses in the magnetic layer which increase with the sensitivity. In fact, it could be shown that the magnetically induced flicker phase noise is directly proportional to the sensitivity, leading to a LOD theoretically being independent of the sensitivity. The determined limits of detection for both modes are depicted in Figure A2b yielding values of $700pT/\sqrt{\mathrm{Hz}}$ (0th mode) and $3nT/\sqrt{\mathrm{Hz}}$, each at an exemplary frequency of 10 Hz. Although the sensitivities of both modes differ by a factor of about 39, the difference in LOD in the flicker noise regime is only a factor of about 4.3. Even if, from a theoretical point of view, an identical LOD could have been expected in principle, the discrepancy can be easily explained. The hysteresis losses, represented by the imaginary part of the magnetic material’s complex permeability, generally increase with higher excitation frequencies approaching ferromagnetic resonance [40,41,42], thus leading to a worse flicker phase noise-to-sensitivity ratio in the higher frequency 1st mode.
